# Low dose naltrexone in multiple sclerosis: Effects on medication use. A quasi-experimental study

**DOI:** 10.1371/journal.pone.0187423

**Published:** 2017-11-03

**Authors:** Guttorm Raknes, Lars Småbrekke

**Affiliations:** 1 Regional Medicines Information and Pharmacovigilance Centre (RELIS), University Hospital of North Norway, Tromsø, Norway; 2 Raknes Research, Ulset, Norway; 3 Department of Pharmacy, Faculty of Health Sciences, UiT—The Arctic University of Norway, Tromsø, Norway; Royal College of Surgeons in Ireland, IRELAND

## Abstract

Low dose naltrexone (LDN) has become a popular off-label therapy for multiple sclerosis (MS). A few small, randomized studies indicate that LDN may have beneficial effects in MS and other autoimmune diseases. If proven efficacious, it would be a cheap and safe alternative to the expensive treatments currently recommended for MS. We investigated whether a sudden increase in LDN use in Norway in 2013 was followed by changes in dispensing of other medications used to treat MS. We performed a quasi-experimental before–and–after study based on population data from the Norwegian Prescription Database (NorPD). We included all patients that collected at least one LDN prescription in 2013, and had collected at least two medications with a reimbursement code for MS, or collected a medication with MS as the only indication in 2009 or 2010. Outcomes were differences in cumulative dispensed doses and incidence of users of disease modifying MS therapies, and medications used to treat MS symptoms two years before and two years after dispensing the initial LDN prescription. The eligible 341 patients collected 20 921 prescriptions in the observation period. Apart from changes in line with general trends in MS therapy in Norway, there was no difference in neither dispensed cumulative doses or number of prevalent users of MS specific medication. Initiation of LDN was not followed by reductions of other medications used to treat symptoms associated with MS.

## Introduction

Naltrexone is an opioid antagonist originally used to treat opioid and alcohol addiction [1,2]. In the past two decades, naltrexone in low doses (<5 mg/day, Low Dose Naltrexone, LDN) has gained popularity among some patients and doctors as off-label treatment of multiple sclerosis (MS) and other autoimmune diseases like Crohn’s disease, psoriasis, and rheumatoid arthritis [3]. Few studies have investigated the efficacy of LDN in MS, hence LDN should be considered an experimental, alternative therapy.

The suggested mechanisms of action for LDN in autoimmune diseases are mainly related to endogenous opioid activity [4]. In MS, one hypothesis is that LDN results in reduced apoptosis in oligodendrocytes [5]. The safety profile of naltrexone is well known when used for approved indications at the standard dose of 50 mg/day. The FDA issued a black box warning concerning hepatotoxicity in high doses (>300 mg/day), but this has not been considered a clinical problem at standard doses [6]. The risk of hepatotoxicity is probably lower for LDN compared to higher doses of naltrexone. Recently, Norwegian neurologists described a case of treatment-resistant immune thrombocytopenic purpura (ITP) that was probably linked to LDN use by a MS patient [7]. However, ITP is also a known adverse reaction of interferon beta, a standard treatment of MS [8].

In Internet forums, a number of MS patients report beneficial effects of LDN including reduced relapse rate and slowed disease progression. They also report fewer side effects than most other established therapies. In August 2017, LDN was rated better than baclofen, glatiramer acetate, intravenous glucocorticoids, and interferons beta as MS therapy on CureTogether, an online social patient network [9].

High quality clinical and pharmacoepidemiological studies evaluating the effect of LDN in MS are lacking, possibly because naltrexone is a low cost generic drug with limited commercial potential. If efficacy is proven comparable to or better than standard treatment options, it could be a cheap alternative to many expensive medicines.

The effects on quality of life (QoL) of LDN in MS have been examined in two placebo-controlled, double-blinded crossover studies. One study funded by American LDN supporters, found significant effects on some QoL measures. A similar study from Iran found no effect in patients receiving 4.5 mg naltrexone/day [10,11]. In a retrospective study on long-term treatment in MS, the authors found that LDN monotherapy did not result in an exacerbation of disease symptoms, adverse effects or deterioration of general health [12].

On February 28 2013, the biggest commercial television station in Norway (TV2) aired a documentary on the alleged beneficial effects of LDN in MS. Patients with severe MS explained how the use of LDN had almost normalized their functioning [13]. This resulted in a substantial increase in the awareness of LDN among patients with a wide range of chronic diseases. According to data from the Norwegian Prescription Database (NorPD), the yearly periodic prevalence of LDN users increased from < 100 to more than 11 000 during 2013 [14]. This sudden increase provided an unprecedented opportunity for quasi-experimental studies to assess the effect of LDN on prescribing of other medicines in MS and other chronic conditions. Using NorPD data, we have already observed that the sudden increase in the use of LDN in Norway in 2013 was followed by a significant reduction in opioid dispensing among persistent LDN users [15].

It is reasonable to assume some correlation between severity of MS symptoms and the type and amount of drugs dispensed to MS patients. If LDN has a clinical effect in MS, it is plausible that initiation of LDN therapy could be followed by detectable changes in the dispensing of disease modifying substances, or changes in dispensing of drugs used to treat MS symptoms (e.g. glucocorticoids and baclofen). However, there is insufficient evidence to conclude any direct association between dispensing of drugs and severity of the disease. Regardless whether LDN has an effect or not in MS, it is interesting to investigate potential changes in prescription patterns of relevant medication following initiation of this controversial treatment. Such changes would affect costs, side effects and drug interactions in this patient group where polypharmacy is common, and should be of interest to both prescribers, patients and those who pay for health services.

### Objective

The aim of this study was to evaluate whether initiation of LDN therapy was followed by a change in the dispensing of medication used in MS. If so, were there any differences between MS patients that collected LDN once, and MS patients that collected LDN twice or more?

## Methods

### Study design/Setting/Resources for the study

We performed a quasi-experimental study based on data from NorPD, which contains individual data on all prescriptions dispensed since 2004 to the entire Norwegian population excluding hospital and nursing home patients. Details on NorPD are published elsewhere [16]. In short, each prescription in NorPD contains a unique pseudonym for the personal identifier and demographic data of both the patient and the prescriber, the medical specialty of the prescriber, the Anatomical Therapeutic Chemical classification (ATC) code and the amount of drug in defined daily dose (DDD), date of dispensing, and location of the dispensing pharmacy. The Norwegian Institute of Public Health is the host of the database [17]. It is possible to follow individual patients’ dispensing over time as NorPD contains information about reimbursed as well as non-reimbursed prescriptions. Only dispensed products that have a product identifying number are recorded, and consequently, products produced in the pharmacy such as reformulated naltrexone tablets are not included. The database contains no information on the indication for therapy, but has disease codes (ICD-10 or ICPC-2) for reimbursement. We applied to NorPD and paid a fee to obtain a data file containing information from January 1 2009 to December 31 2015 for all Norwegian patients that had collected at least one LDN prescription (product identification code 361181) in 2013 [18].

### Study subjects

MS patients who had collected at least one LDN prescription in 2013 were included in the study, and we defined the first LDN dispensing date as “Index date”.

Patients who in 2009 and 2010 had collected medication that is only approved for MS or with a reimbursement code for MS (ICD-10 G35 or ICPC-2 N86) were considered MS patients. Patients who collected naltrexone before 2013 and patients who died before 2013 were excluded.

We stratified all MS patients into three groups based on the number of LDN prescriptions dispensed during 2013 and 2014:

$$\begin{array}{l} LDN\;x\;1\;(one\;time\;users):Collected\;only\;one\;LDN\;prescription\\ LDN\;x\;2–3:Collected\;two\;or\;three\;LDN\;prescriptions\\ LDN\;x\;4+\;(persistent\;users):Collected\;four\;or\;more\;LDN\;prescriptions \end{array}$$

We have previously shown that median naltrexone daily dose among Norwegian persistent LDN users in 2013 and 2014 was 3.7 mg [14]. A majority of patients in the LDN x 4+ group collected LDN sufficient for at least one whole year’s continuous use, and they were classified as persistent users. The patients in the LDN x 1 group presumably only had LDN available for a short period, and could be considered a control group.

### Variables

We used the following variables in this study: person identifier for patient, patient age and sex, reimbursement code, ATC code, product identifying number, date of dispensing, and dispensed volume in DDDs.

### Outcome variables

The primary outcome measure was a change in the dispensing of disease modifying agents as well as baclofen and systemic glucocorticoids in each group (LDN x 1, LDN x 2–3, LDN x 4+). We analyzed all disease modifying agents separately and aggregated in drug classes. In order to account for new treatment options during the study period, we performed pooled analyses on agents that had a secular reduction in dispensing in the Norwegian population (interferon beta and glatiramer acetate), and on disease modifying MS agents (dimethyl fumarate, fampridin, fingolimod, teriflunomide) that were introduced on the Norwegian market during the observation period. Secondary outcomes were changes in medications indirectly associated with disease activity or quality of life for MS patients. MS patients frequently report pain, and we therefore focused on analgesics [18]. We also considered drugs used for urinary frequency and incontinence, constipation and erectile dysfunction in addition to antidepressants, hypnotics, and benzodiazepines. A detailed description of outcomes is given in S1 Table. All outcomes were expressed as differences in average cumulative DDD in each group, and as a change in number of users as percentage of all patients in each group between the two years (730 days) preceding and the two years following the Index date.

### Measurement

For each group, we added up the number of average DDDs collected and the number of users for each drug in the two years (730 days) before and two years after the Index date (Index date + 729 days). This means that the total observation time was four years, and the first potential outcome observation date before Index date was January 1 2011, and the last potential observation date after Index date was December 31 2015.

### Bias

To increase the specificity of the MS diagnosis, patients had to have collected at least two prescriptions that met inclusion criteria (see S1 Text for complete inclusion criteria). We assumed that newly diagnosed MS patients would have a larger increase in MS-specific drugs than patients that were observed over the entire period. Inclusion was therefore based on NorPD data from the two (2009 and 2010) years preceding the observation period (2011–2015).

### Study size

The number of patients fulfilling our inclusion criteria in NorPD determined study size.

### Statistical methods

Data were prepared for analyses in SPSS 23 and Excel 2013. Pairwise two-sided t-test was used to test significance of mean changes in DDD in each group for all examined drugs. Confidence intervals (95%) for difference of means were calculated. Change of number of users was expressed as proportion (%) of each group with confidence interval (95%) calculated in accordance with method for non-independent proportions [19]. A significance level of 0.05 was used to assess whether there were changes in prescribing before and after LDN different from zero, or difference-in-difference between groups.

### Ethics

The project protocol was submitted to the Regional Committee for Medical and Health Research Ethics of Northern Norway. The committee concluded that disclosure was not mandatory, since the data were pseudonymized. The local privacy ombudsman for research at the University Hospital of North Norway approved the project. For Norwegian central health registers like NorPD, consent from individual patients is by law not required. A significance level of 0.05 was used to assess whether there were changes in prescribing before and after LDN different from zero, or difference-in-difference between groups.

## Results

### Participants

**Fig 1:**
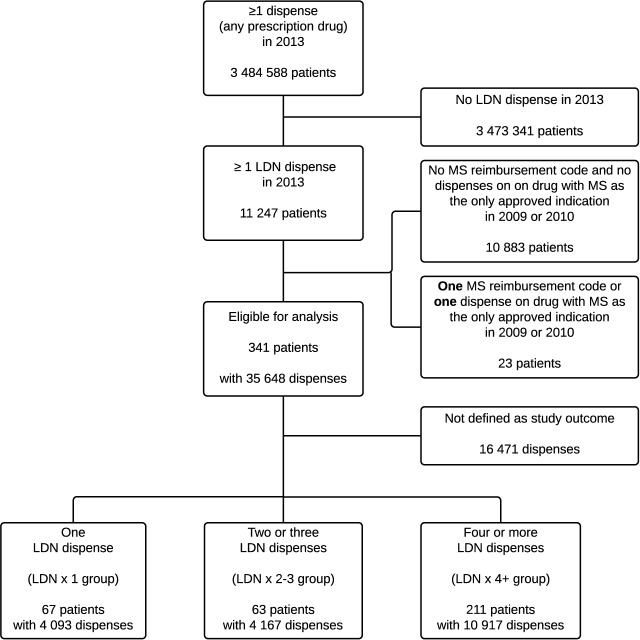
Flowchart of the inclusion of patients and dispensed medications.

The inclusion of patients and the number of dispensed medications of interest are presented in Fig 1. All prescriptions collected by the included patients in the entire observation period (two years before and two years after the Index date) were available for analyses, in total 16 368 patient months. Baseline characteristics are presented in Table 1. The proportion of females was highest in the LDN x 2–3 group.

**Table 1:** Baseline data of the three cohorts with standard error (SE). Total dispenses per user includes outcome and non-outcome drugs 2 years before LDN.

		LDN x 1	LDN x 2–3	LDN x 4+
**N**		**67**	**63**	**211**
**Age (mean± SE)**		45.2 (± 1.3)	48.4 (± 1.3)	48.8 (± 0.6)
**Females (% ± SE)**		45 (67.2 ± 5.7)	50 (79.4 ± 5.1)	150 (71.1 ± 3.1)
**Total dispenses per user (± SE)**		28.7 (± 4.1)	35.3 (± 7.5)	29.0 (± 1.8)
**Users (% ± SE) 2 years before LDN of:**				
	**Disease modifying MS drugs**	56 (83.6 ± 4.5)	48 (76.2 ± 5.4)	169 (80.1 ± 2.7)
	**Baclofen**	11 (16.4 ± 4.5)	13 (20.6 ± 5.1)	53 (25.1 ± 3.0)
	**Systemic glucocorticoids**	9 (13.4 ± 4.2)	8 (12.7 ± 4.2)	21 (10,0 ± 2.1)
	**Antidepressants, benzodiazepines or Z hypnotics**	26 (38.8 ± 6.0)	32 (50.8 ± 6.3)	79 (37.4 ± 3.3)

The included patients collected 1 744 LDN prescriptions in the study period, and the median was 5 dispensed LDN prescriptions. There were 10 261 dispensed prescriptions of outcome drugs two years before Index date, and 8 916 two years after.

**Fig 2:**
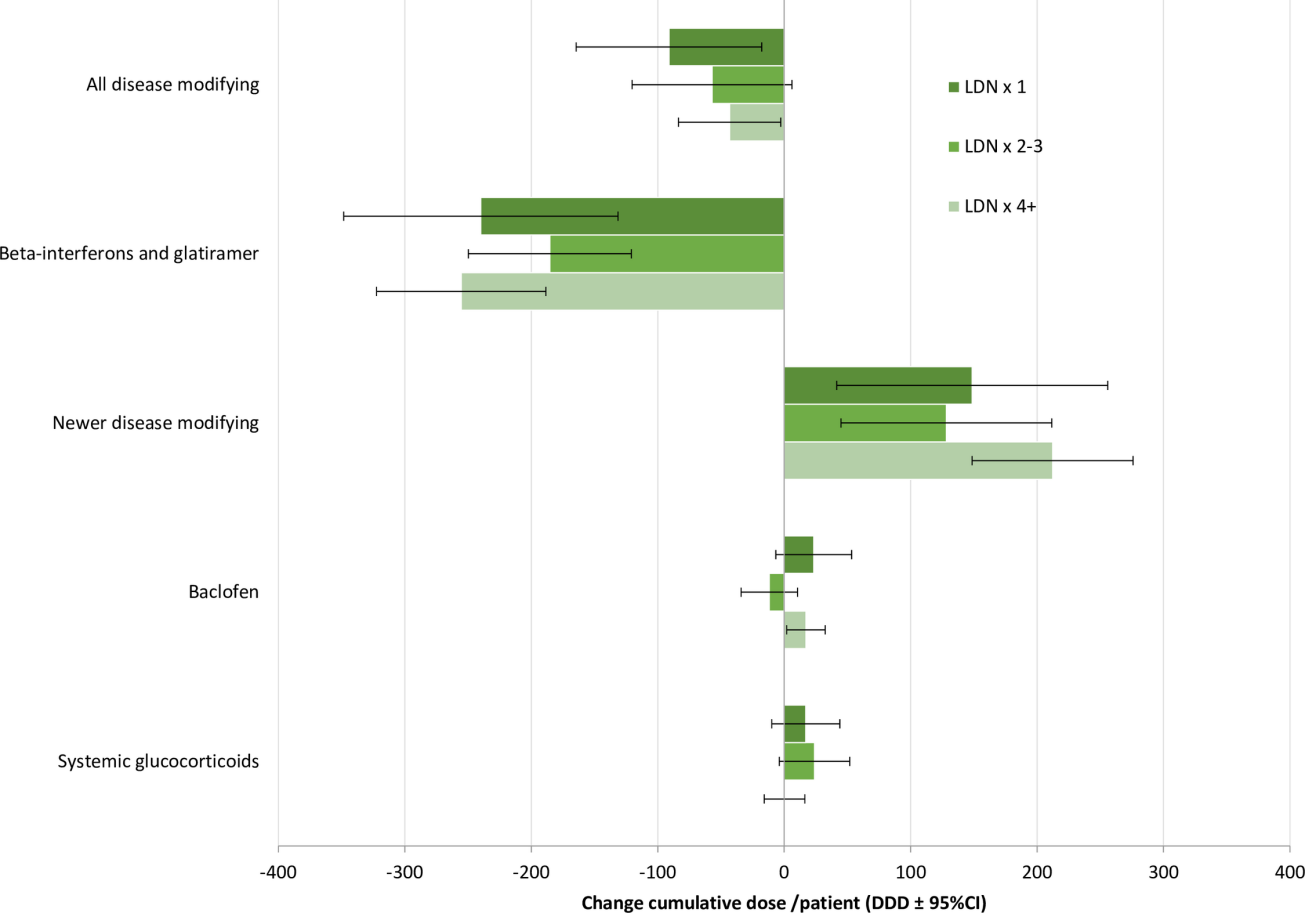
Difference in dispensing of disease modifying MS agents, baclofen and systemic glucocorticoid after initiation of LDN therapy by number of LDN dispenses. Newer disease modifying MS agents include fampridin, fingolimod, teriflunomide and dimethyl fumarate. Cumulative dose in DDD per patient two years prior to Index date compared to two years after.

Changes in the dispensing of disease modifying MS drugs, systemic glucocorticoids, and baclofen are presented in Fig 2, Table 2 (DDDs), and in S2 Table and S1 Fig (number of users). Changes in the other drugs included in the analysis are shown in Table 3 (DDDs) and in S3 Table (number of users). There was no significant difference between the groups of LDN users. There was a significant reduction in the dispensing of interferons and glatiramer acetate in all groups, both in terms of total cumulative DDDs and number of prevalent users. Contrary to this, there was a significant increase in the dispensing of newer disease modifying MS drugs in all groups. When combining all disease modifying drugs, we observed a significant reduction of users and cumulative DDDs among the persistent LDN users. There was a similar tendency in the other groups, but it was only statistically significant for cumulative DDDs among one-time users of LDN. The number of baclofen users increased significantly in the LDN x 1 group, and the cumulative baclofen DDDs increased significantly in the LDN x 4+ group. There was no significant change in systemic glucocorticoid dispensing. Opioid dispensing was significantly reduced in persistent LDN users, where the cumulative dose was down by 42% and the number of users was down by 9%. In addition, we observed a significant reduction in users of NSAIDs (-8%) and an increase in users of other analgesics and antipyretics in this group. No other changes in the dispensing of pain relievers were observed. Cannabinoid dispensing increased significantly among persistent LDN users, both in terms of DDDs and in number of users. In this group, there was also a significant increase (22%) in Z-hypnotic DDDs, and an increase in users of incontinency drugs. There were no significant changes in the dispensing of antidepressants, benzodiazepines, drugs used in constipation, or erectile dysfunction following LDN therapy. In the LDN x 1 group, gabapentin DDDs decreased significantly, and the proportion of pregabalin users increased after LDN use.

**Table 2:** Difference in use of disease modifying MS drugs, systemic glucocorticoids and baclofen two years before and after first LDN prescription by number of LDN dispenses to patient.

			Average DDD per patient				
	ATC-code	Group	Before LDN	After LDN	Difference of mean	(95% CI)	p
**Interferon beta-1a**	L03 B07	*LDN x 1*	262.1	144.9	-117.2	(-223.5 to -10.9)	0.031
		*LDN x 2–3*	237.4	144.1	-93.4	(-149.6 to -37.2)	0.002
		*LDN x 4+*	245.4	101.2	-144.2	(-210.3 to -78.0)	<0.001
**Interferon beta-1b**	L03 B08	*LDN x 1*	37.1	13.4	-23.6	(-43.4 to -3.9)	0.020
		*LDN x 2–3*	16.0	14.3	-1.7	(-11.0 to 7.6)	0.713
		*LDN x 4+*	29.7	8.5	-21.2	(-35.2 to -7.2)	0.003
**Glatiramer acetate**	L03 X13	*LDN x 1*	149.2	50.1	-99.0	(-147.7 to -50.4)	<0.001
		*LDN x 2–3*	190.2	100.0	-90.2	(-135.3 to -45.1)	<0.001
		*LDN x 4+*	153.0	62.9	-90.1	(-115.9 to -64.3)	<0.001
**Fingolimod**	L04A A27	*LDN x 1*	38.7	81.1	42.4	(9.0 to 75.9)	0.014
		*LDN x 2–3*	16.3	26.3	10.0	(-11.2 to 31.2)	0.350
		*LDN x 4+*	41.7	94.8	53.0	(28.1 to 78.0)	<0.001
**Teriflunomide**	L04A A31	*LDN x 1*	0.0	50.1	50.1	(12.7 to 87.6)	0.009
		*LDN x 2–3*	0.0	49.3	49.3	(14.1 to 84.5)	0.007
		*LDN x 4+*	0.0	36.5	36.5	(19.3 to 53.6)	<0.001
**Fampridin**	N07X X07	*LDN x 1*	105.3	116.0	10.7	(-41.6 to 62.9)	0.685
		*LDN x 2–3*	105.3	128.0	22.7	(-10.2 to 55.5)	0.173
		*LDN x 4+*	151.8	226.2	74.4	(48.3 to 100.5)	<0.001
**Dimethyl fumarate**	N07X X09	*LDN x 1*	0.0	45.6	45.6	(13.4 to 77.7)	0.006
		*LDN x 2–3*	0.0	46.3	46.3	(14.9 to 77.6)	0.004
		*LDN x 4+*	0.0	48.4	48.4	(30.7 to 66.0)	<0.001
**Systemic glucocorticoids**	H02A B	*LDN x 1*	9.7	26.9	17.1	(-9.8 to 44.0)	0.208
		*LDN x 2–3*	18.8	42.9	24.0	(-3.8 to 51.9)	0.090
		*LDN x 4+*	17.7	18.0	0.3	(-15.8 to 16.4)	0.970
**Baclofen**	M03B X01	*LDN x 1*	70.9	94.3	23.4	(-6.6 to 53.3)	0.124
		*LDN x 2–3*	82.9	71.1	-11.7	(-34.1 to 10.6)	0.170
		*LDN x 4+*	72.0	89.2	17.2	(2.0 to 32.5)	0.027

Mean cumulative dose in defined daily doses (DDD) per patient two years prior to Index date compared to two years after.

**Table 3:** Difference in use of drugs used to treat MS symptoms in defined daily doses (DDD) two years before and after initiation of LDN therapy by number of LDN dispenses to patient.

			Average DDD per user				
	ATC-code	Group	Before	After	Difference of mean	95% CI	p
**Antibiotics**	J01	*LDN x 1*	20.4	24.6	4.2	(-7.1 to 15.5)	0.462
		*LDN x 2–3*	33.7	29.1	-4.6	(-22.6 to 13.3)	0.607
		*LDN x 4+*	27.9	26.2	-1.7	(-9 to 5.6)	0.647
**Drugs for urinary frequency and incontinence**	G04B D	*LDN x 1*	160.9	132.2	-28.6	(-82.4 to 25.2)	0.292
		*LDN x 2–3*	175.6	162.7	-12.9	(-66 to 40.3)	0.630
		*LDN x 4+*	213.8	237.7	23.9	(-10.8 to 58.6)	0.176
**Drugs used for constipation**	A06	*LDN x 1*	18.8	23.1	4.3	(-1.1 to 9.8)	0.119
		*LDN x 2–3*	80.8	55.4	-25.4	(-66 to 15.1)	0.215
		*LDN x 4+*	44.9	48.0	3.2	(-14.9 to 21.3)	0.727
**Drugs used in erectile dysfunction**	G04 BE	*LDN x 1*	22.0	28.3	6.4	(-8.6 to 21.3)	0.400
		*LDN x 2–3*	2.8	4.0	1.2	(-1 to 3.4)	0.286
		*LDN x 4+*	9.3	11.3	2.0	(-1.4 to 5.5)	0.248
**Methenamine**	J01X X05	*LDN x 1*	68.3	68.1	-0.2	(-21.7 to 21.2)	0.984
		*LDN x 2–3*	57.6	57.8	0.2	(-14.5 to 14.8)	0.981
		*LDN x 4+*	63.2	65.6	2.3	(-8.9 to 13.6)	0.682
**Benzodiazepine related drugs** (Z-hypnotics)	N05C F	*LDN x 1*	105.7	91.9	-13.8	(-89 to 61.5)	0.716
		*LDN x 2–3*	214.2	216.1	1.9	(-58.5 to 62.2)	0.951
		*LDN x 4+*	114.3	139.0	24.8	(6.5 to 43)	0.008
**Benzodiazepines**	N05 CD	*LDN x 1*	5.2	15.7	10.5	(-10.4 to 31.3)	0.319
		*LDN x 2–3*	4.0	2.7	-1.3	(-4.5 to 2)	0.437
		*LDN x 4+*	8.6	13.0	4.4	(-5 to 13.8)	0.354
**Antidepressants (except TCA)**	N06A	*LDN x 1*	130.6	154.8	24.2	(-20.6 to 68.9)	0.285
		*LDN x 2–3*	213.7	210.8	-2.9	(-56 to 50.1)	0.913
		*LDN x 4+*	136.9	139.8	2.9	(-21.1 to 26.9)	0.813
**Opioids**	N02A	*LDN x 1*	79.4	47.8	-31.6	(-111 to 47.9)	0.430
		*LDN x 2–3*	30.4	40.0	9.7	(-7.6 to 26.9)	0.267
		*LDN x 4+*	48.3	28.2	-20.1	(-31.6 to -8.6)	0.001
**Other analgesics and antipyretics**	N02B	*LDN x 1*	33.4	41.3	8.0	(-9.2 to 25.1)	0.357
		*LDN x 2–3*	86.8	62.0	-24.8	(-62.4 to 12.8)	0.192
		*LDN x 4+*	40.8	48.3	7.5	(-3.1 to 18.1)	0.165
**NSAIDs**	M01A	*LDN x 1*	48.2	41.4	-6.8	(-19.1 to 5.4)	0.267
		*LDN x 2–3*	76.8	87.8	11.0	(-37.2 to 59.2)	0.650
		*LDN x 4+*	76.4	66.1	-10.3	(-27.8 to 7.2)	0.246
**Tricyclic antidepressants**	N06A A	*LDN x 1*	25.8	29.6	3.7	(-4.9 to 12.3)	0.388
		*LDN x 2–3*	39.1	44.2	5.1	(-9.1 to 19.3)	0.476
		*LDN x 4+*	18.8	17.3	-1.5	(-6.2 to 3.3)	0.547
**Gabapentin**	N03A X12	*LDN x 1*	54.2	35.0	-19.2	(-45.1 to 6.7)	0.144
		*LDN x 2–3*	38.5	14.6	-24.0	(-57.5 to 9.6)	0.158
		*LDN x 4+*	53.1	57.2	4.1	(-15.8 to 24)	0.687
**Pregabalin**	N03A X16	*LDN x 1*	51.4	125.2	73.8	(5.2 to 142.4)	0.035
		*LDN x 2–3*	60.2	49.2	-11.0	(-52.5 to 30.5)	0.598
		*LDN x 4+*	40.5	39.9	-0.6	(-15.1 to 13.9)	0.935
**Cannabinoids**	N02B G10	*LDN x 1*	1.7	6.7	5.0	(-1.5 to 11.6)	0.129
		*LDN x 2–3*	5.0	6.0	1.0	(-8 to 9.9)	0.827
		*LDN x 4+*	5.3	25.9	20.6	(7.8 to 33.5)	0.002

Mean cumulative dose in defined daily doses (DDD) per patient two years prior to Index date compared to two years after.

## Discussion

Apart from general trends in prescribing of MS drugs in Norway, we observed no changes in dispensing to MS patients following initiation of LDN therapy. There were no significant difference-in-difference between groups, indicating no association between LDN exposure and changes in dispensing to MS patients.

### Interpretation

The observed differences in MS medications are probably explained by changes in recommendations of MS therapy implemented during the observation period. For example, for interferon beta, both the number of users and the total collected numbers of DDDs were reduced by nearly 50% in all LDN groups. This coincides with a decrease in dispensing of interferon beta in Norway from 2012 to 2015 [20]. The observed reduction in the number of users of interferon-beta and glatiramer acetate that were dispensed and the increase in natalizumab, fingolimod, fampridin, and dimethyl fumarate dispensing also coincides with total dispensing in Norway (see S2 Fig) [20].

It is important to emphasize that this study is only able to detect differences in prescription patterns, it cannot prove or reject efficacy of LDN in MS. It is possible that patients continued their previous medication despite significant clinical improvement caused by LDN, or because it was helpful for symptoms that LDN did not alleviate. Possibly, the patients may have continued the treatment solely because of their doctors’ recommendation. We believe this is an unlikely explanation, since patients by law are required to be informed and involved in decisions about diagnosis and treatment [21]. Hypothetically, if LDN had striking effects, for example by leading to complete recovery from MS, it is plausible that changes in dispensing would have been detectable. In that case we would also expect larger changes in dispensing in persistent LDN users compared to one-time users. However, we were unable to identify any significant difference between the groups in terms of neither MS specific drugs nor other relevant medications. The observed significant increase in the dispensing of baclofen, cannabinoids, and drugs used for urinary frequency and incontinence among the persistent LDN users further weakens the assumption that LDN is effective against a range of MS symptoms. Opioid dispensing was reduced in MS patient with persistent LDN use, but this was in the same order of magnitude as seen in the entire persistent LDN using population [15].

### Strengths and limitations

This is the first pharmacoepidemiological study on LDN in MS, and the study is based on data covering the entire Norwegian population. A major strength is that we followed dispensing patterns of individuals in defined groups over five years allowing a quasi-experimental study where the initiation of LDN therapy could be considered an intervention in a natural experiment. The number of included patients is higher than in previous studies on LDN in MS. However, NorPD has several important limitations. Except from information on dispensing, there is no validated clinical information. We therefore had to identify MS patients indirectly. Our inclusion criteria were strict, and we are confident that the included persons had been MS patients for some years. On the other hand, we lost LDN-using MS patients that did not collect prescriptions with MS specific medication or reimbursement codes in 2009 or 2010, and our conclusions cannot be extrapolated to newly diagnosed patients. We also lost any medication administered directly from hospitals, and it is likely that changes in drug exposure during the most severe relapses were not captured by NorPD. LDN was not included in NorPD before 2013, and it was impossible to identify patients that used LDN before the only registered LDN product got an identification number in NorPD on May 15 2013. An unknown, but probably low number of MS patients who were using LDN before 2013 were thus likely to have been included in this study. We find it unlikely that inclusion of these patients could explain why we were unable to identify changes in prescribing indicating beneficial effects of LDN. It is questionable to what extent changes in dispensing of baclofen and systemic glucocorticoids are relevant outcomes, as only a minority of the included patients collected these medicines. The use of systemic glucocorticoids could also be associated with other conditions than MS. Preferably, we should have included a control group consisting of MS patients who did not use LDN. We believe, however, that the group with one-time users of LDN is a good substitute since these patients did not use LDN for most of the observation period.

The results are presented in a before-and-after manner, and there is a risk of bias due to secular trends. To account for this, we have performed a post hoc interrupted time series analyses for the primary outcomes (see S2 Text, S3 and S4 Figs and S4–S7 Tables). This did not reveal any difference in difference in dispensing between groups.

### Generalizability

Although this study was performed on the Norwegian population under extraordinary conditions when an unprecedented wave of LDN use hit in 2013, we believe it was a unique opportunity for pharmacoepidemiological studies on the efficacy of LDN in MS. The quasi-experimental design, with observation both before and after initiating LDN, and in three groups with different LDN exposure should be able to capture relevant changes in dispense patterns caused by initiation of LDN.

## Conclusion

Initiation of LDN therapy did not lead to a reduction in the dispensing of other MS medications. Randomized clinical studies are needed to determine whether LDN has any beneficial effects in MS or not.

## Supporting information

S1 Fig Difference in dispensing of disease modifying MS agents, baclofen and systemic glucocorticoid after initiation of LDN therapy. Newer disease modifying MS agents include fampridin, fingolimod, teriflunomide and dimethyl fumarate. Percent change in number of users as proportion of entire group two years prior to Index date compared to two years after.
(PDF)

S2 Fig Trends in prevalent users of baclofen, systemic glucocorticoids, newer disease modifying MS agents and interferon beta / glatiramer acetate 2011–2015 in the entire Norwegian population. Newer disease modifying MS agents include fampridin, fingolimod, teriflunomide and dimethyl fumarate.
(PDF)

S3 Fig Sum of defined daily doses (DDDs) per patient of disease modifying MS agents in 30 days intervals. Interrupted time series two years before and after first low dose naltrexone (LDN) dispense (time = 0) in three groups with different LDN exposure.
(PDF)

S4 Fig Sum of defined daily doses (DDDs) per patient of baclofen in 30 days intervals. Interrupted time series two years before and after first low dose naltrexone (LDN) dispense (time = 0) in three groups with different LDN exposure.
(PDF)

S1 Table Overview of primary and secondary outcomes. Outcomes were change in average number of cumulative defined daily doses (DDDs) per user and change in number of users of each drug.
(PDF)

S2 Table Change in number of users of disease modifying MS drugs, systemic glucocorticoids and baclofen two years before and after first LDN prescription by number of LDN dispenses. Change in prevalent users as proportion (%) of entire group ± 95% confidence interval.
(PDF)

S3 Table Change in number of users of drugs used to treat MS symptoms two years before and after first LDN prescription by number of LDN dispenses. Change in prevalent users as proportion (%) of entire group ± 95% confidence interval.
(PDF)

S4 Table Interrupted time series, disease modifying MS agents. Difference in slope (coefficient) and intercept two years before and two years after first low dose naltrexone (LDN) dispense. Sum of DDD/patient in 30 days intervals in three groups with different LDN exposure.
(PDF)

S5 Table Interrupted time series, disease modifying MS agents, comparison of groups. Difference in difference of slope (coefficient) and intercept two years before and two years after first low dose naltrexone (LDN) dispense. Sum of DDD/patient in 30 days intervals in three groups with different LDN exposure.
(PDF)

S6 Table Interrupted time series, baclofen. Difference in slope (coefficient) and intercept two years before and two years after first low dose naltrexone (LDN) dispense. Sum of DDD/patient in 30 days intervals in three groups with different LDN exposure.
(PDF)

S7 Table Interrupted time series, baclofen, comparison of groups. Difference in difference of slope (coefficient) and intercept two years before and two years after first low dose naltrexone (LDN) dispense. Sum of DDD/patient in 30 days intervals in three groups with different LDN exposure.
(PDF)

S1 Text Inclusion criteria details. (PDF)

S2 Text Summary of interrupted time series (ITS) analysis. (PDF)
