# Supplementary figures and images for: Low dose naltrexone in multiple sclerosis: Effects on medication use. A quasi-experimental study
Source: PLoS One. 2017 Nov 3;12(11):e0187423. doi: 10.1371/journal.pone.0187423 (PMC5669439; doi:10.1371/journal.pone.0187423)

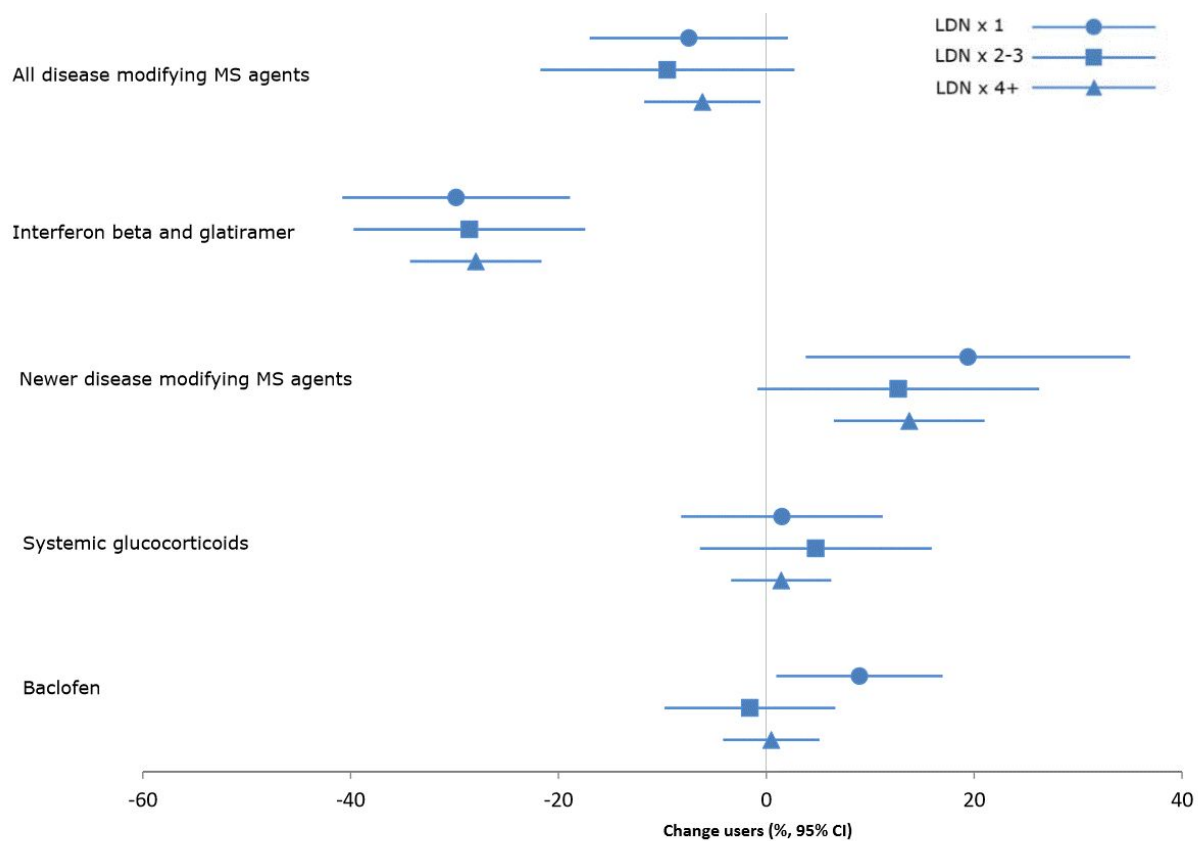

Supplement: S1 Fig — Newer disease modifying MS agents include fampridin, fingolimod, teriflunomide and dimethyl fumarate. Percent change in number of users as proportion of entire group two years prior to Index date compared to two years after. (PDF) [file pone.0187423.s001.pdf]
