# Supplementary material for: Low dose naltrexone in multiple sclerosis: Effects on medication use. A quasi-experimental study
Source: PLoS One. 2017 Nov 3;12(11):e0187423. doi: 10.1371/journal.pone.0187423 (PMC5669439; doi:10.1371/journal.pone.0187423)

S2 Figure. Trends in prevalent users of baclofen, systemic glucocorticoids, newer disease modifying MS agents and interferon beta / glatiramer acetate 2011-2015 in the entire Norwegian population. Newer disease modifying MS agents include fampridin, fingolimod, teriflunomide and dimethyl fumarate.

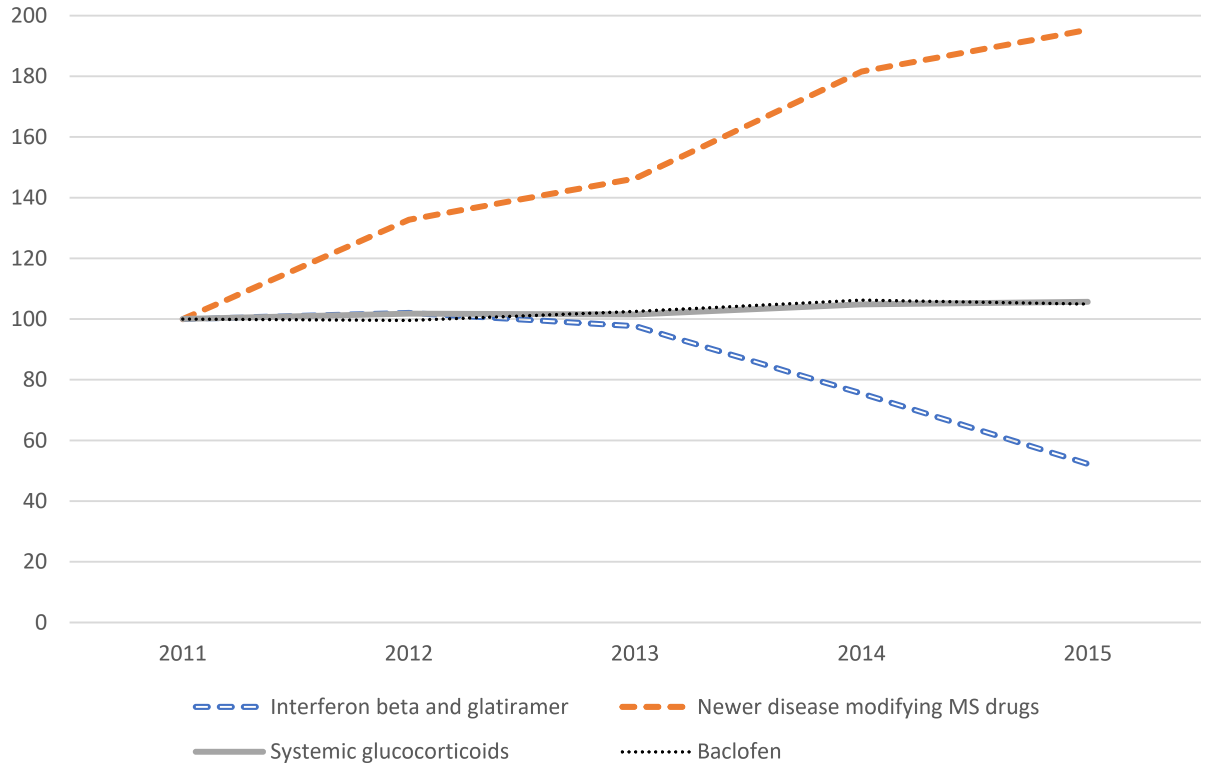

Supplement: S2 Fig — Newer disease modifying MS agents include fampridin, fingolimod, teriflunomide and dimethyl fumarate. (PDF) [file pone.0187423.s002.pdf]
