# Supplementary material for: Low dose naltrexone in multiple sclerosis: Effects on medication use. A quasi-experimental study
Source: PLoS One. 2017 Nov 3;12(11):e0187423. doi: 10.1371/journal.pone.0187423 (PMC5669439; doi:10.1371/journal.pone.0187423)

S3 Figure. Sum of defined daily doses (DDDs) per patient of disease modifying MS agents in 30 days intervals. Interrupted time series two years before and after first low dose naltrexone (LDN) dispense (time=0) in three groups with different LDN exposure.

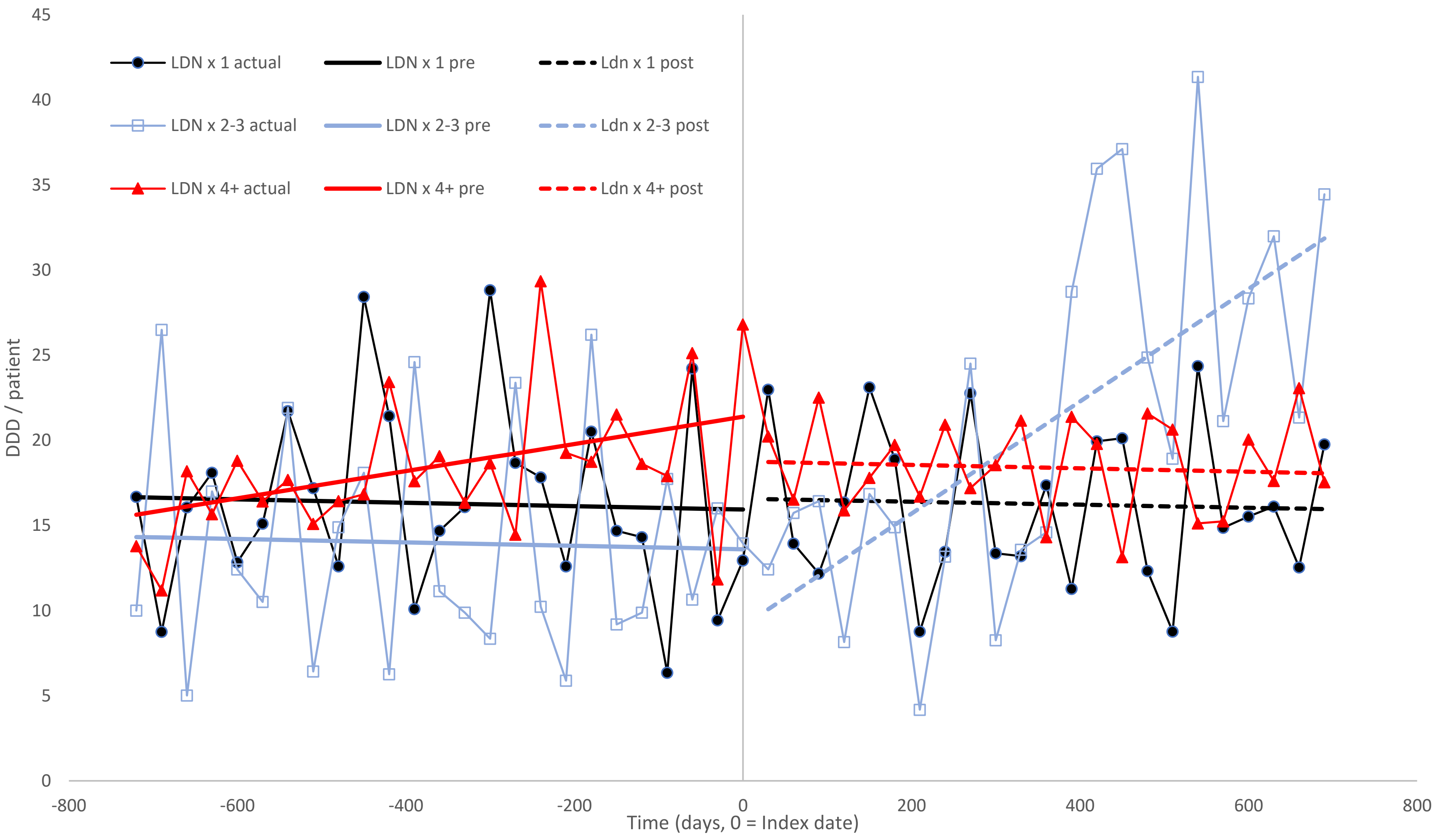

Supplement: S3 Fig — Interrupted time series two years before and after first low dose naltrexone (LDN) dispense (time = 0) in three groups with different LDN exposure. (PDF) [file pone.0187423.s003.pdf]
