# Supplementary material for: Low dose naltrexone in multiple sclerosis: Effects on medication use. A quasi-experimental study
Source: PLoS One. 2017 Nov 3;12(11):e0187423. doi: 10.1371/journal.pone.0187423 (PMC5669439; doi:10.1371/journal.pone.0187423)

S4 Figure. Sum of defined daily doses (DDDs) per patient of baclofen in 30 days intervals. Interrupted time series two years before and after first low dose naltrexone (LDN) dispense (time=0) in three groups with different LDN exposure.

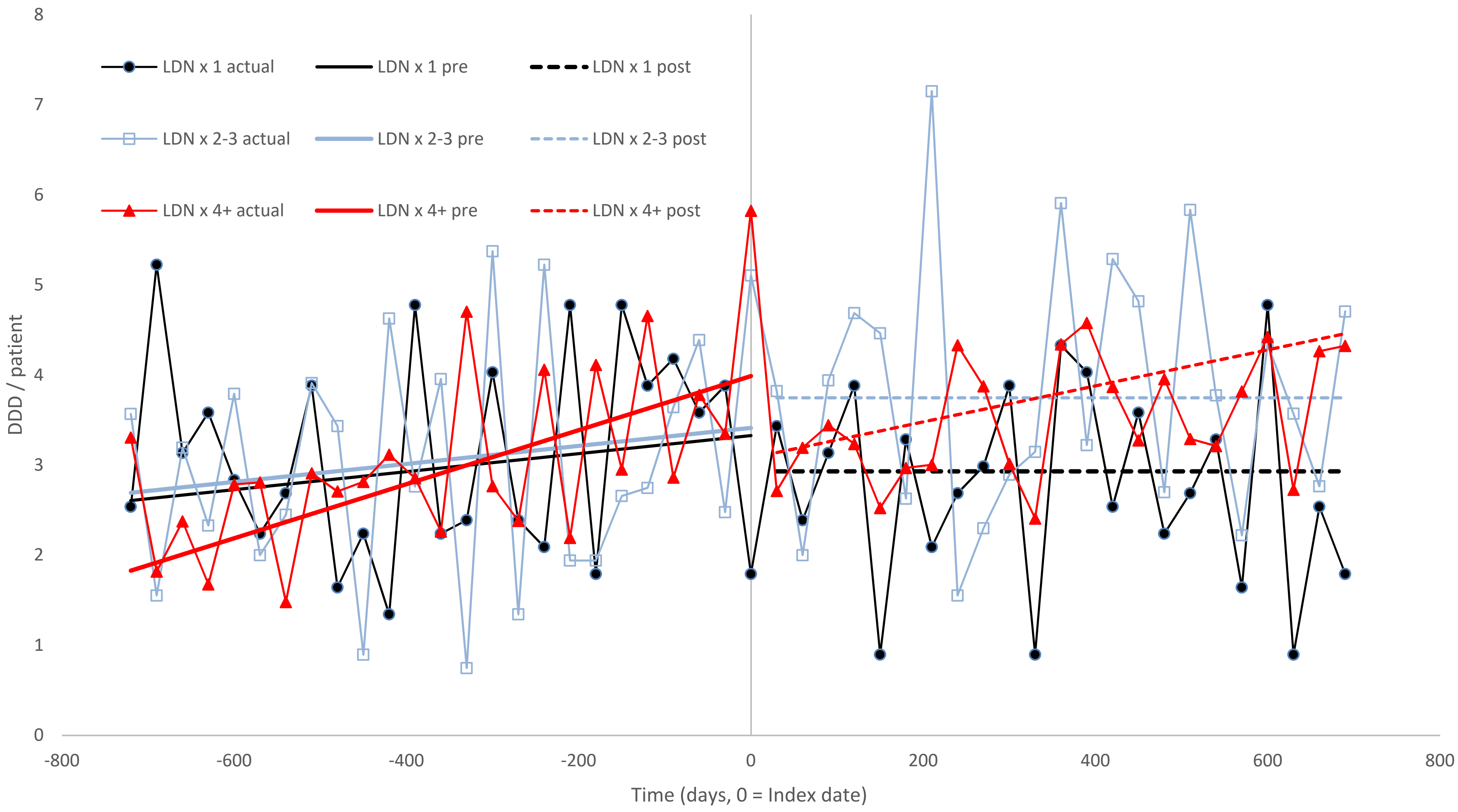

Supplement: S4 Fig — Interrupted time series two years before and after first low dose naltrexone (LDN) dispense (time = 0) in three groups with different LDN exposure. (PDF) [file pone.0187423.s004.pdf]
