# Supplementary material for: Low dose naltrexone in multiple sclerosis: Effects on medication use. A quasi-experimental study
Source: PLoS One. 2017 Nov 3;12(11):e0187423. doi: 10.1371/journal.pone.0187423 (PMC5669439; doi:10.1371/journal.pone.0187423)
Supplement: S1 Table — Outcomes were change in average number of cumulative defined daily doses (DDDs) per user and change in number of users of each drug. (PDF) [file pone.0187423.s005.pdf]

S1 Table. Overview of primary and secondary outcomes. Outcomes were change in average number of cumulative defined daily doses (DDDs) per user and change in number of users of each drug.

Primary outcomes		Secondary outcomes	
**ATC code**	**Drug/drug groups**	**ATC code**	**Drug/drug groups**
**Betainterferons and glatiramer acetate**		**G04B D**	Drugs for urinary frequency and incontinence
**L03A B07**	Interferon beta 1a	**A06**	Drugs used for constipation
**L03A B08**	Interferon beta 1b	**G04B E**	Drugs used in erectile dysfunction
**L03A X13**	Glatiramer acetate	**N05C F**	Z-hypnotics
		**N05C D**	Benzodiazepines
**Newer MS disease modifying agents:**		**N02A**	Opioids
**L04A A27**	Fingolimod	**N02B**	Other analgesics and antipyretics (excluding cannabinoids)
**L04A A31**	Teriflunomide	**M01A**	Non steroid antiinflammatory drugs (NSAIDs)
**L07X X07**	Fampridin	**N06A A**	Tricyclic antidepressants
**L07X X09**	Dimethyl fumarate	**N06A**	All other antidepressants
		**N03A X12**	Gabapentin
**H02A B**	Systemic glucocorticoids	**N03A X16**	Pregabalin
**M03B X01**	Baclofen	**N02B G10**	Cannabinoids
