# Supplementary material for: Low dose naltrexone in multiple sclerosis: Effects on medication use. A quasi-experimental study
Source: PLoS One. 2017 Nov 3;12(11):e0187423. doi: 10.1371/journal.pone.0187423 (PMC5669439; doi:10.1371/journal.pone.0187423)
Supplement: S2 Table — Change in prevalent users as proportion (%) of entire group ± 95% confidence interval. (PDF) [file pone.0187423.s006.pdf]

S2 Table. Change in number of users of disease modifying MS drugs, systemic glucocorticoids and baclofen two years before and after first LDN prescription by number of LDN dispenses. Three groups based on number of LDN dispenses: LDN x 1 (N=67) collected LDN once, LDN x 2-3 (N=63) two or three times, and LDN x 4+ (N=211) four or more times.

			Users before LDN		Users after LDN		Change prevalent users		
	**ATC-code**	**Cohort**	**N**	**%**	**N**	**%**	**%**	**95 % CI**	**p**
**Interferon beta-1a**	L03 B07	*LDN x1*	14	20.9	8	11.9	-9.0	(-15.8 to -2.1)	0.015
		LDN x 2-3	14	22.2	6	9.5	-12.7	(-20.9 to -4.5)	0.004
		LDN x 4+	47	22.3	22	10.4	-11.8	(-16.2 to -7.5)	<0.001
**Interferon beta-1b**	L03 B08	*LDN x1*	6	9.0	2	3.0	-6.0	(-11.6 to -0.3)	0.048
		LDN x 2+	2	3.2	1	1.6	-1.6	(-4.7 to 1.5)	0.240
		LDN x 4+	13	6.2	4	1.9	-4.3	(-7.0 to -1.5)	0.004
**Glatiramer acetate**	L03A X13	*LDN x1*	27	40.3	14	20.9	-19.4	(-28.9 to -9.9)	<0.001
		LDN x 2-3	22	34.9	13	20.6	-14.3	(-22.9 to -5.6)	0.002
		LDN x 4+	68	32.2	38	18.0	-14.2	(-19.5 to -9.0)	<0.001
**Fingolimod**	L04A A27	*LDN x1*	7	10.4	10	14.9	4.5	(-3.2 to 12.1)	0.207
		LDN x 2-3	6	9.5	6	9.5	0.0	(-7.6 to 7.6)	0.399
		LDN x 4+	21	10.0	40	19.0	9.0	(4.3 to 13.7)	<0.001
**Teriflunomid**	L04A A31	*LDN x1*	0	0.0	7	10.4	10.4	(3.1 to 17.8)	0.008
		LDN x 2-3	0	0.0	9	14.3	14.3	(5.6 to 22.9)	0.002
		LDN x 4+	0	0.0	23	10.9	10.9	(6.7 to 15.1)	<0.001
**Fampridin**	N07X X07	*LDN x1*	21	31.3	16	23.9	-7.5	(-18.7 to 3.7)	0.170
		LDN x 2-3	18	28.6	17	27.0	-1.6	(-9.8 to 6.6)	0.371
		LDN x 4+	95	45.0	79	37.4	-7.6	(-12.7 to -2.4)	0.006
**Dimethyl fumarate**	N07X X09	*LDN x1*	0	0.0	10	14.9	14.9	(6.4 to 23.5)	0.001
		LDN x 2-3	0	0.0	11	17.5	17.5	(8.1 to 26.8)	0.001
		LDN x 4+	0	0.0	31	14.7	14.7	(9.9 to 19.5)	<0.001
**Systemic glucocorticoids**	H02A B	*LDN x1*	9	13.4	10	14.9	1.5	(-8.2 to 11.2)	0.381
		LDN x 2-3	8	12.7	11	17.5	4.8	(-6.4 to 15.9)	0.281
		LDN x 4+	21	10.0	24	11.4	1.4	(-3.4 to 6.2)	0.338
**Baclofen**	M03B X01	*LDN x1*	11	16.4	17	25.4	9.0	(1.0 to 17.0)	0.036
		LDN x 2-3	13	20.6	12	19.0	-1.6	(-9.8 to 6.6)	0.371
		LDN x 4+	53	25.1	54	25.6	0.5	(-4.2 to 5.1)	0.391

Change in prevalent users as proportion (%) of entire group ± 95 % confidence interval.
