# Supplementary material for: Low dose naltrexone in multiple sclerosis: Effects on medication use. A quasi-experimental study
Source: PLoS One. 2017 Nov 3;12(11):e0187423. doi: 10.1371/journal.pone.0187423 (PMC5669439; doi:10.1371/journal.pone.0187423)
Supplement: S3 Table — Change in prevalent users as proportion (%) of entire group ± 95% confidence interval. (PDF) [file pone.0187423.s007.pdf]

S3 Table. Change in number of users of drugs used to treat MS symptoms two years before and after first LDN prescription by number of LDN dispenses. Three groups based on number of LDN dispenses: LDN x 1 (N=67) collected LDN once, LDN x 2-3 (N=63) two or three times and LDN x 4+ (N=211) four or more times.

			Users before LDN		Users after LDN		Change prevalent users		
	**ATC code**	**Group**	**N**	**(%)**	**N**	**(%)**	**%**	**95% CI**	**p**
**Antibiotics**	J01	*LDN x1*	40	59.7	40	59.7	0.0	(-14.3 to 14.3)	0.399
		LDN x 2-3	34	54.0	37	58.7	4.8	(-12.0 to 21.5)	0.341
		LDN x 4+	117	55.5	116	55.0	-0.5	(-8.4 to 7.5)	0.396
**Drugs for urinary frequency and incontinence**	G04B D	*LDN x1*	17	25.4	14	20.9	-4.5	(-10.9 to 2.0)	0.158
		LDN x 2-3	17	27.0	15	23.8	-3.2	(-11.9 to 5.6)	0.310
		LDN x 4+	53	25.1	65	30.8	5.7	(1.0 to 10.4)	0.023
**Drugs used for constipation**	A06	*LDN x1*	4	6.0	6	9.0	3.0	(-4.1 to 10.1)	0.285
		LDN x 2-3	6	9.5	7	11.1	1.6	(-8.7 to 12.0)	0.381
		LDN x 4+	14	6.6	17	8.1	1.4	(-1.7 to 4.5)	0.265
**Drugs used in erectile dysfunction**	G04 BE	*LDN x1*	8	11.9	10	14.9	3.0	(-1.1 to 7.1)	0.142
		LDN x 2-3	5	7.9	4	6.3	-1.6	(-4.7 to 1.5)	0.240
		LDN x 4+	23	10.9	19	9.0	-1.9	(-4.8 to 1.0)	0.178
**Methenamine**	J01X X05	*LDN x1*	9	13.4	11	16.4	3.0	(-2.8 to 8.8)	0.240
		LDN x 2-3	9	14.3	10	15.9	1.6	(-5.4 to 8.5)	0.361
		LDN x 4+	32	15.2	31	14.7	-0.5	(-3.8 to 2.9)	0.384
**Benzodiazepine related drugs**	N05C F	*LDN x1*	20	29.9	25	37.3	7.5	(-1.1 to 16.1)	0.094
		LDN x 2-3	21	33.3	23	36.5	3.2	(-6.6 to 13.0)	0.326
		LDN x 4+	58	27.5	63	29.9	2.4	(-1.9 to 6.6)	0.219
**Benzodiazepines**	N05 CD	*LDN x1*	1	1.5	2	3.0	1.5	(-1.4 to 4.4)	0.240
		LDN x 2-3	2	3.2	2	3.2	0.0	(-4.4 to 4.4)	0.399
		LDN x 4+	4	1.9	2	0.9	-0.9	(-2.3 to 0.4)	0.145
**Antidepressants (except TCA)**	N06A	*LDN x1*	13	19.4	14	20.9	1.5	(-6.2 to 9.2)	0.371
		LDN x 2-3	18	28.6	22	34.9	6.3	(-2.3 to 15.0)	0.142
		LDN x 4+	46	21.8	44	20.9	-0.9	(-5.5 to 3.6)	0.367
**Opioids**	N02A	*LDN x1*	24	35.8	31	46.3	10.4	(-4.0 to 24.9)	0.145
		LDN x 2-3	19	30.2	26	41.3	11.1	(-1.4 to 23.6)	0.088
		LDN x 4+	69	32.7	50	23.7	-9.0	(-16.0 to -2.0)	0.017
**Other analgesics and antipyretics**	N02B	*LDN x1*	20	29.9	24	35.8	6.0	(-3.2 to 15.1)	0.176
		LDN x 2-3	29	46.0	25	39.7	-6.3	(-17.0 to 4.3)	0.202
		LDN x 4+	61	28.9	74	35.1	6.2	(0.9 to 11.4)	0.029
**NSAIDs**	M01A	*LDN x1*	33	49.3	30	44.8	-4.5	(-17.2 to 8.2)	0.314
		LDN x 2-3	30	47.6	30	47.6	0.0	(-13.2 to 13.2)	0.399
		LDN x 4+	104	49.3	87	41.2	-8.1	(-15.0 to -1.1)	0.030
**Tricyclic antidepressants**	N06A A	*LDN x1*	12	17.9	10	14.9	-3.0	(-10.1 to 4.1)	0.285
		LDN x 2-3	10	15.9	11	17.5	1.6	(-6.6 to 9.8)	0.371
		LDN x 4+	21	10.0	21	10.0	0.0	(-4.4 to 4.4)	0.399
**Gabapentin**	N03A X12	*LDN x1*	16	23.9	8	11.9	-11.9	(-20.7 to -3.1)	0.012
		LDN x 2-3	8	12.7	6	9.5	-3.2	(-11.9 to 5.6)	0.310
		LDN x 4+	41	19.4	37	17.5	-1.9	(-7.1 to 3.4)	0.311
**Pregabalin**	N03A X16	*LDN x1*	10	14.9	10	14.9	0.0	(-8.3 to 8.3)	0.399
		LDN x 2-3	7	11.1	10	15.9	4.8	(-2.1 to 11.6)	0.158
		LDN x 4+	20	9.5	17	8.1	-1.4	(-5.3 to 2.4)	0.306
**Cannabinoids**	N02B G10	*LDN x1*	3	4.5	5	7.5	3.0	(-2.8 to 8.8)	0.240
		LDN x 2-3	3	4.8	4	6.3	1.6	(-3.8 to 7.0)	0.337
		LDN x 4+	11	5.2	22	10.4	5.2	(1.5 to 9.0)	0.010

Change in prevalent users as proportion (%) of entire group ± 95 % confidence interval.
