# Supplementary material for: Low dose naltrexone in multiple sclerosis: Effects on medication use. A quasi-experimental study
Source: PLoS One. 2017 Nov 3;12(11):e0187423. doi: 10.1371/journal.pone.0187423 (PMC5669439; doi:10.1371/journal.pone.0187423)
Supplement: S4 Table — Difference in slope (coefficient) and intercept two years before and two years after first low dose naltrexone (LDN) dispense. Sum of DDD/patient in 30 days intervals in three groups with different LDN exposure. (PDF) [file pone.0187423.s008.pdf]

S4 Table. Interrupted time series, disease modifying MS agents

	Slope (x 10^{-3}) (95% CI)		p	Intercept (95% CI)		p
**LDN x 1**	0.1	(-1.2 to 12.4)	0.985	0.63	(-4.54 to 5.80)	0.811
**LDN x 2-3**	34.3	(18.6 to 49.9)	<0.001	-4.51	(-11.11 to 2.10)	0.182
**LDN x 4+**	-8.9	(-14.9 to -3.0)	0.004	-2.63	(-5.16 to -0.11)	0.042

. Difference in slope (coefficient) and intercept two years before and two years after first low dose naltrexone (LDN) dispense. Sum of DDD/patient in 30 days intervals in three groups with different LDN exposure.
