# Supplementary material for: Low dose naltrexone in multiple sclerosis: Effects on medication use. A quasi-experimental study
Source: PLoS One. 2017 Nov 3;12(11):e0187423. doi: 10.1371/journal.pone.0187423 (PMC5669439; doi:10.1371/journal.pone.0187423)
Supplement: S5 Table — Difference in difference of slope (coefficient) and intercept two years before and two years after first low dose naltrexone (LDN) dispense. Sum of DDD/patient in 30 days intervals in three groups with different LDN exposure. (PDF) [file pone.0187423.s009.pdf]

S5 Table. Interrupted time series, disease modifying MS agents, comparison of groups.

	Slope (x 10^{-3}) (95% CI)		p	Intercept (95% CI)		p
**LDN x 1 vs. LDN x 2-3**	34.2	(14.3 to 54.0)	0.001	-5.1	(-13.5 to 3.3)	0.234
**LDN x 1 vs. LDN x 4+**	-9.0	(-22.7 to 4.6)	0.199	-3.3	(-9.0 to 2.5)	0.271
**LDN x 2-3 vs. LDN x 4+**	-43.2	(-59.9 to -26.5)	<0.001	1.9	(-5.2 to 9.0)	0.604

Difference in difference of slope (coefficient) and intercept two years before and two years after first low dose naltrexone (LDN) dispense. Sum of DDD/patient in 30 days intervals in three groups with different LDN exposure.
