# Supplementary material for: Low dose naltrexone in multiple sclerosis: Effects on medication use. A quasi-experimental study
Source: PLoS One. 2017 Nov 3;12(11):e0187423. doi: 10.1371/journal.pone.0187423 (PMC5669439; doi:10.1371/journal.pone.0187423)
Supplement: S6 Table — Difference in slope (coefficient) and intercept two years before and two years after first low dose naltrexone (LDN) dispense. Sum of DDD/patient in 30 days intervals in three groups with different LDN exposure. (PDF) [file pone.0187423.s010.pdf]

S6 Table. 6: Interrupted time series, baclofen.

	Slope (x 10^{-3}) (95% CI)		p	Intercept (95% CI)		p
**LDN x 1**	-1.0	(-3.1 to 1.1)	0.371	0.40	(-.50 to 1.29)	0.387
**LDN x 2-3**	-0.9	(-3.3 to 1.5)	0.460	0.33	(-0.67 to 1.35)	0.518
**LDN x 4+**	-1.4	(-2.8 to 0.0)	0.047	-0.91	(-1.50 to -0.32)	0.003

Difference in slope (coefficient) and intercept two years before and two years after first low dose naltrexone (LDN) dispense. Sum of DDD/patient in 30 days intervals in three groups with different LDN exposure.
