# Supplementary material for: Low dose naltrexone in multiple sclerosis: Effects on medication use. A quasi-experimental study
Source: PLoS One. 2017 Nov 3;12(11):e0187423. doi: 10.1371/journal.pone.0187423 (PMC5669439; doi:10.1371/journal.pone.0187423)
Supplement: S7 Table — Difference in difference of slope (coefficient) and intercept two years before and two years after first low dose naltrexone (LDN) dispense. Sum of DDD/patient in 30 days intervals in three groups with different LDN exposure. (PDF) [file pone.0187423.s011.pdf]

S7 Table. Interrupted time series, baclofen, comparison of groups.

	Slope (x 10^{-3}) (95% CI)		p	Intercept (95% CI)		p
**LDN x 1 vs. LDN x 2-3**	0.1	(-3.1 to 3.2)	0.968	-0.06	(-1.41 to 1.29)	0.929
**LDN x 1 vs. LDN x 4+**	-0.5	(-3.0 to 2.1)	0.727	-1.30	(-2.38 to -0.23)	0.020
**LDN x 2-3 vs. LDN x 4+**	-0.5	(-3.3 to 2.2)	0.714	-1.24	(-2.41 to -0.07)	0.039

Difference in difference of slope (coefficient) and intercept two years before and two years after first low dose naltrexone (LDN) dispense. Sum of DDD/patient in 30 days intervals in three groups with different LDN exposure.
