# Supplementary material for: Low dose naltrexone in multiple sclerosis: Effects on medication use. A quasi-experimental study
Source: PLoS One. 2017 Nov 3;12(11):e0187423. doi: 10.1371/journal.pone.0187423 (PMC5669439; doi:10.1371/journal.pone.0187423)
Supplement: S1 Text — (PDF) [file pone.0187423.s012.pdf]

S1 Text: Inclusion criteria details.

1. Collected at least one LDN prescription (NorPD product identification code 361181) in 2013.

AND

2.
    a. Collected at least two prescriptions with reimbursement code for multiple sclerosis in 2009 OR 2010

        i. ICD-10: G35

        OR

        ii. ICPC-2: N86

OR

    b. Collected at least two prescriptions with ATC-codes medication with MS as the only approved indication in 2009 OR 2010

        i. L03A B07 Interferon beta-1a
        ii. L03A B08 Interferon beta-1b
        iii. L03A X13 Glatiramer acetate
        iv. L04A A27 Fingolimod
        v. L04A A31 Teriflunomide
        vi. N07X X07 Fampridin
        vii. N07X X09 Dimethyl fumarate
