# Supplementary material for: Low dose naltrexone in multiple sclerosis: Effects on medication use. A quasi-experimental study
Source: PLoS One. 2017 Nov 3;12(11):e0187423. doi: 10.1371/journal.pone.0187423 (PMC5669439; doi:10.1371/journal.pone.0187423)
Supplement: S2 Text — (PDF) [file pone.0187423.s013.pdf]

S2 Text. Summary of interrupted time series (ITS) analysis.

Background

We used a quasi-experimental before-and-after design when analysing the outcomes of this study. An important limitation in a before-after study is that findings may be biased by trends and cyclical effects. To account for this, we reanalysed defined daily doses (DDDs) per patient of disease modifying agents, baclofen and systemic glucocorticoids using interrupted time series analyses (ITS).

Method

We performed an ITS on the main outcomes of this study using an autoregressive integrated moving average (ARIMA) model on the sum of dispensed defined daily doses (DDDs) per patient in each group in 30 days intervals. We carried out modeling and statistical tests using SPSS 23 and Excel.

Results

A summary of ITS for disease modifying MS agents is presented in S4 Table and S5 Table and is plotted in S3 Fig. There was no change in the LDN x 1 group. LDN x 2 had a significant increase in slope, and there was a reduction in slope and a borderline significant reduction in intercept among persistent LDN users (LDN x 4+). There was no *difference in difference* between one-time (LDN x 1), and persistent (LDN x 4+) users, but the LDN x 2-3 group had a significant increase in slope compared to the other groups.

ITS for baclofen is summarised in S6 Table, S7 Table and is plotted in S4 Fig. Except for a reduction in intercept and a borderline significant reduction in slope in the LDN x 4+ group, there was no difference in slope or intercept. There was no *difference in difference* between groups.

There were too few dispenses to perform ITS analysis on systemic glucocorticoids, even after aggregating dispenses in intervals up to 100 days.

Interpretation

The ITS analyses did not reveal any findings contradicting the conclusions of the before-and-after analyses. For disease modifying MS agents we identified a significant increase in the LDN x 2-3 group, but this result is in conflict with the hypothesis that LDN is efficacious in MS. This anomaly was probably caused by unknown characteristics of these patients.

A limitation of the ITS in our study design is that the time dimension is centred around the Index date. A consequence of this is a bias due to a high number of dispenses at Index date. To account for this, we repeated the ITS analyses excluding the first 30 day period in which Index date was included (data not shown). This did not change the results of the analyses.
